# Asynchronous Teledermatology for Non-Scarring Alopecia: A Retrospective Study

**DOI:** 10.1177/26924366251366793

**Published:** 2025-08-11

**Authors:** Aliyyat Afolabi, Elijah Brown, Edra K. Ha, Joseph C. English

**Affiliations:** ^1^ School of Medicine, University of Pittsburgh, Pittsburgh, Pennsylvania, USA.; ^2^ Medical Center Department of Dermatology, University of Pittsburgh, Wexford, Pennsylvania, USA.

**Keywords:** alopecia areata, androgenetic alopecia, asynchronous consultations, health care disparities, non-scarring alopecia, traction alopecia, teledermatology, telogen effluvium

## Abstract

**Background:**

Non-scarring alopecia, including androgenetic alopecia (AGA), alopecia areata (AA), telogen effluvium (TE), and traction alopecia (TA), significantly impacts psychosocial well-being. Access to specialized dermatologic care for these conditions is often limited, particularly in underserved populations. Asynchronous teledermatology has emerged as a potential solution to extend care to these groups.

**Objective:**

To evaluate the diagnostic utility and treatment patterns of asynchronous teledermatology for non-scarring alopecia and examine its role in improving care access across diverse populations within the University of Pittsburgh Medical Center (UPMC) network.

**Methods:**

A retrospective study of 321 asynchronous teledermatology cases of non-scarring alopecia from 2022 to 2023 was conducted using the UPMC medical record system. Diagnosist, treatment type, and demographic data were analyzed. Longitudinal outcomes and adherence data were not consistently available.

**Results:**

AA was the most common diagnosis (59.5%), followed by AGA (26.5%), TE (7.5%), and TA (5.0%). A definitive diagnosis was made remotely in 91.3% of cases; only 8.7% required in-person follow-up. Treatment included over-the-counter therapies such as minoxidil and clobetasol, with prescription medications used for moderate to severe cases. Racial demographics reflected high engagement from Black (22.7%) and Asian (12.9%) patients, with 41.7% of patients residing outside Pittsburgh.

**Conclusion:**

Asynchronous teledermatology is an effective tool for diagnosing and managing non-scarring alopecia, facilitating timely intervention and improving access to dermatologic care. Future studies should access patient satisfaction, long-term outcomes, and implementation strategies to further expand equitable teledermatology access.

## Introduction

Non-scarring alopecia encompasses a spectrum of common dermatological conditions, including androgenetic alopecia (AGA), alopecia areata (AA), telogen effluvium (TE), and traction alopecia (TA). These conditions, while not life-threatening, can cause significant psychological distress, including reduced self-esteem, anxiety, and depression.^1,2^ This impact is particularly profound in individuals for whom hair plays a vital role in cultural identity and self-image. Despite the prevalence and emotional toll, non-scarring alopecia are often undertreated due to barriers in accessing specialized dermatologic care. Underserved populations—especially those in rural areas or with limited financial resources—experience delays in diagnosis and treatment, exacerbating disease burden.^3,4^

Teledermatology has emerged as a scalable approach to addressing these gaps in care. Asynchronous teledermatology, where clinical images and a patient’s history are submitted for later review offers notable advantages in flexibility, triage, and reach.^5^ However, limited data exist on its use specifically for diagnosing and managing non-scarring alopecia.

This study evaluates the diagnostic utility, treatment patterns, and demographic reach of asynchronous teledermatology for non-scarring alopecia across the University of Pittsburgh Medical Center (UPMC) network.

## Methods

We conducted a retrospective chart review of 321 patients diagnosed with non-scarring alopecia between 2022 and 2023 through the asynchronous teledermatology platform (e-Derm [patient to physician] and e-Consult [physician to physician]) at UPMC. Referring providers submitted clinical histories and photographs for dermatologist review. Diagnoses were categorized as AGA, AA, TE, TA, or other based on standard clinical criteria.^6,7^

Individualized management plans included pharmacologic recommendations and diagnostic workups such as laboratory tests or scalp biopsies when necessary. Demographic information, including age, sex, race, and geographic location, was collected. Treatment interventions were categorized as over-the-counter (OTC), prescription, educational materials, or other recommendations. Data on treatment adherence and long-term outcomes were not consistently documented. This study was approved by the University of Pittsburgh Institutional Review Board.

## Results

Among 321 patients, the most common diagnosis was AA (59.5%), followed by AGA (26.5%), TE (7.5%), and TA (5.0%). Diagnoses differed significantly between the 2 years studied (*p* = 0.000003) (Table 1).

**Table 1.** Primary Hair Loss Patterns and Appointment Settings for Patients Diagnosed with Non-Scarring Alopecia in 2022 and 2023

	Patients in 2022 (*n* = 154)	Patients in 2023 (*n* = 167)
Primary hair loss pattern		
Alopecia/areata	114	77
Traction alopecia	5	11
AGA	22	63
TE	12	12
Other	1	5
Appointment Setting		
e-Derm	146	157
e-Consult: Outpatient	7	10
Inpatient	1	0

The cohort was racially and geography diverse: 22.7% Black, 56.9% White, 12.9% Asian, and 0.9% Hispanic patients. Compared to the demographics of the UPMC catchment area, Asian patients were significantly overrepresented (12.9% vs. 5.6%, *p* < 0.001), while White (56.9% vs. 64.5%, *p* = 0.006) and Hispanic patients (0.9% vs. 3.6%, *p* = 0.006) were significantly underrepresented. Black patients representation closely mirrored the regional population (22.7% vs. 23.2%, *p* = 0.895). Additionally, 41.7% of patients resided outside Pittsburgh (Table 2).

**Table 2.** Demographic Characteristics of Patients Diagnosed with Non-Scarring Alopecia in 2022 and 2023

Characteristics	Patients in 2022 (*n* = 154) (%)	Patients in 2023 (*n* = 167) (%)
Demographics		
Age, mean (SD)	39.2 ± (14.2)	37.3 ± (13.2)
Gender		
Male	40.26	45.51
Female	59.74	54.49
Race/ethnicity		
NH White	53.25	60.48
NH Black	22.72	22.75
NH Asian	15.58	10.18
Hispanic	1.23	0.599
Other/unreported	4.54	5.39

A definitive diagnosis was rendered remotely in 91.3% of cases. The remaining 8.7% required in-person evaluation, often for scalp biopsy or further diagnostic clarification.

Treatment approaches varied based on diagnosis and disease severity. OTC therapies such as minoxidil, clobetasol, and low-level light therapy were commonly recommended. For patients with severe AA, intralesional corticosteroids were administered during in-person visits (12.5%). Providers also uploaded educational materials to improve patient understanding of their condition and treatment options. Relevant laboratory tests, including iron panels, thyroid function tests, hormonal assessments, and vitamin level evaluations, were ordered when indicated to guide personalized management.

Treatment strategies included OTC therapies (e.g., topical minoxidil, clobetasol, and low-level light therapy), prescription medications (e.g., oral corticosteroids, immunosuppressants, and anti-androgens), and educational materials. Most patients (92.5%) received educational materials to support treatment understanding. “Other” recommendations included lifestyle counseling, in-person procedures, or declined interventions (Table 3).

**Table 3.** Frequency of Treatment Modalities by Alopecia Subtype

Alopecia subtype	OTC treatments (%)	Prescription treatments (%)	Education materials (%)	Other recommendations (%)
Alopecia/Areata	86 (45.0%)	58 (30.4%)	173 (90.6%)	51 (26.7%)
Androgenetic Alopecia (AGA)	69 (81.2%)	31 (36.5%)	81 (95.3%)	7 (8.2%)
Telogen Effluvium (TE)	13 (54.2%)	2 (8.3%)	24 (100%)	7 (29.2%)
Traction Alopecia (TA)	9 (56.3%)	3 (18.8%)	15 (93.8%)	3 (18.8%)
Other	0 (0%)	4 (80%)	3 (60%)	1 (20%)

## Discussion

This study demonstrates the effectiveness of asynchronous teledermatology in diagnosing and managing non-scarring alopecia, especially in racially and geographically diverse populations. With over 91% of cases managed remotely, the model supports timely triage and treatment without necessitating physical appointments for most patients.^8^

These findings align with prior studies demonstrating strong diagnostic reliability of teledermatology across dermatologic conditions.^5,8^ For instance, Bourkas et al. compared teledermatology and face-to-face (F2F) agreement in primary diagnoses in 2023.^8^ The study reviewed 44 articles and found a pooled agreement rate of 68.9%.^8^ Ultimately, the meta-analysis did not reveal a significant difference between teledermatology and F2F consultations.^8^ While variations in technology and physician training may have influenced outcomes, the authors concluded that teledermatology remains an excellent resource for patients with limited access to dermatology clinics.^7,8^ Rapid turnaround (typically within 24–48 h) may mitigate disease progression and reduce wait times.^3^ The engagement of Black and Asian patients further suggests the model’s potential to address disparities in dermatologic care. A 2024 study examined the rise in melanoma incidence among Hispanic individuals by conducting five focus groups with a total of 34 participants to explore contributing factors.^9^ Niu et al. identified the top three perceived barriers as low levels of knowledge, low prioritization, and limited access to care.^9^ While this may seem counterintuitive, the authors recommended focusing on community- and system-level strategies to enhance access, which could also help address similar barriers faced by patients with alopecia.

Treatment patterns reflected evidence-based practice, with OTC minoxidil and clobetasol as first-line recommendations.^10^ Table 3 summarizes treatment modalities by alopecia subtype for clarity. Psychological implications of alopecia should also not be overlooked, particularly as these conditions can severely impact health-related quality of life.^1,4^ In 2019, Vélez-Muñiz et al. conducted a cross-sectional study with 126 participants over a 1-year period.^1^ Only 25.4% were children, with ages ranging from 3 to 78 years. Using the Dermatology Life Quality Index score, they found that 76.7% of children with AA and 77.6% of adults reported a decreased quality of life associated with feelings of shame about hair loss.^1^ This study was useful in highlighting the connection between AA and the deterioriation in patients’ quality of life.

However, several limitations exist. Patient satisfaction, treatment adherence, and longitudinal outcomes were not assessed. Future studies should incorporate structured follow-up and outcome measurement to evaluate the clinical effectiveness and patient perception of asynchronous care.

## Conclusion

Asynchronous teledermatology is a feasible and effective model for managing non-scarring alopecia. It offers timely, equitable access to dermatologic care across diverse populations. To enhance its utility, future research should include patient-reported outcomes, adherence tracking, and cost-effectiveness analyses.

## Abbreviations Used

AA: Alopecia areata
AGA: Androgenetic alopecia
OTC: Over the counter
TA: Traction Alopecia
TE: Telogen effluvium
UPMC: University of Pittsburgh Medical Center
